# Simulation of the Hybrid Carbon-Aramid Composite Materials Based on Mechanical Characterization by Digital Image Correlation Method

**DOI:** 10.3390/polym13234184

**Published:** 2021-11-29

**Authors:** Camelia Cerbu, Stefania Ursache, Marius Florin Botis, Anton Hadăr

**Affiliations:** 1 Department of Mechanical Engineering, Faculty of Mechanical Engineering, Transilvania University of Brasov, B-dul Eroilor, No. 29, 500036 Brasov, Romania; cerbu@unitbv.ro (C.C.); stefania.olareanu@unitbv.ro (S.U.); 2 Department of Civil Engineering, Faculty of Civil Engineering, Transilvania University of Brasov, B-dul Eroilor, No. 29, 500036 Brasov, Romania; 3 Department of Strength of Materials, Faculty of Industrial Engineering and Robotics, Politehnica University of Bucharest, 313 Splaiul Independentei, 060042 Bucharest, Romania; anton.hadar@upb.ro

**Keywords:** carbon fibres, aramid fibres, Kevlar, hybrid composite, digital image correlation method, tensile test, Poisson ratio, bending test, Charpy test

## Abstract

As hybrid carbon-aramid composites become widely used in various industries, it has become imperative to mechanically characterize them using accurate methods of measuring the entire deformation field such as the digital image correlation (DIC) method. The accuracy of the numerical simulation of carbon-aramid composite structures depends on the accuracy of the elastic constants. Therefore, the goal of this research is to model and simulate the mechanical behaviour of the composite based on epoxy resin reinforced with carbon-aramid woven fabric by considering the mechanical properties investigated by tensile test combined with DIC and the bending test. The curves of the transverse strains related to the longitudinal strains were investigated using DIC in order to determine the Poisson’s ratios in the case of tensile tests applied in warp or weft directions of the reinforcement fabric. The impact strength determined by Charpy tests is also reported. The other main objective is to use the analytical models to compute the tensile and flexural moduli of elasticity for the fictitious orthotropic materials which behave similarly to the carbon-aramid composite investigated. The simulations regarding the behaviour of the carbon-aramid composite in tensile and bending tests were validated by the experimental results, since the maximum errors recorded between experimental and theoretical results were 0.19% and 0.15% for the equivalent tensile modulus and for the equivalent flexural modulus, respectively.

## 1. Introduction

The utilization of natural or synthetic fibres for manufacturing of the composite materials has found significant applications in a variety of fields, such as construction, the automotive industry, the aerospace industry, the shipbuilding industry and the biomedical industry [1,2,3,4]. Composites reinforced with carbon fibre or carbon–Kevlar hybrid fabrics are widely used in the aerospace and automotive industries due to their high performance properties in terms of high strength-to-weight and stiffness-to-weight ratios, as well as their impact resistance in the case of composites reinforced additionally with Kevlar fibres [5,6].

Parts made of composite materials reinforced with carbon or carbon-aramid fabrics, used in the aerospace industry and in the automotive industry, must be designed and manufactured with high precision [5,6]. As a result, it is necessary to determine with great precision the mechanical characteristics of such materials by using precise, fast measurement methods such as the digital image correlation (DIC) method. Considering the elastic properties of carbon-aramid composite materials, the states of stresses and deformations are frequently simulated in the design stage of the structures made of such materials. Consequently, it becomes imperative to use analytical calculation models and numerical models to simulate the mechanical behaviour of structures made of carbon-aramid composites.

Synthetic fibres (carbon Kevlar, glass, Kevlar aramid fibres, basalt) offer excellent strength and stiffness to the composite materials, making them more practical towards load-bearing applications in composite structures [7,8,9]. Although, the carbon fibre reinforced polymers (CFRP) are known for their high strength-to-weight and stiffness-to-weight ratios [10], solutions have been found for the continuous improvement of their properties by adding the carbon nanofibres to reduce thermal residual stress [11]. Other advantages of the carbon-reinforced composites include: long fatigue life; low density; corrosion resistance; wear resistance; environmental stability. There are a lot of applications of the carbon composites in aerospace industry [5]. A recent study [6] showed that the carbon fibres can be used in various parts of vehicle, such as in the bumpers, due to providing an improvement in strength and an appreciable reduction in the weight and size of the frame.

Taking into account the automotive applications for carbon composite structures which sometimes are dynamically loaded, solutions were sought for hybridization of the carbon composites with Kevlar fibres [12,13], or even with natural fibres (jute fibres) [14].

The aramid fibres are organic fibres belonging to the aromatic polyamides, and exhibit high strength, high flexibility, high modulus, low elongation, low density, reduced thermal conductivity, corrosion resistance and absorb significant amounts of energy in impact loading [15,16]. The aramid fibres have different applications, such as bullet-proof jackets; body armour has the capability to stop a bullet fired from a 0.45 calibre gun but rarely stops anything more powerful, for example, a bullet of 7.62 mm which is fired from an AK 47 assault rifle [16,17].

Hybrid composite materials have extensive engineering applications where a high strength to weight ratio, low cost and ease of fabrication are required. They provide improved properties such as tensile modulus, flexural strength and impact strength [12,14,18,19].

Regarding the hybridization mechanism between different fibres, Xian et al. showed in a recent study [20,21] that the hybrid mechanism also affects the performance evolution of the carbon-glass fibre composite rods exposed to freezing-thawing and outdoor environments. This research revealed that the temperature-alternating leads to the degradation of the shear strength at interface and the strength retention is 38.7%. Li et al. [22] evaluated the effects of the hybridization mechanism between carbon and glass fibres on the long-term fatigue behaviour of the pultruded carbon-glass hybrid rod exposed to elevated temperature and hydraulic pressure.

Based upon recent studies, it was concluded that hybridization of carbon fibres with aramid fibres improves the mechanical behaviour of the hybrid composite in impact loading and reduces the post-impact strength losses compared to the carbon/epoxy composites [12,13,23].

In the traditional design of composite structures, the properties of the composite materials are routinely estimated and simulated with well-recognized models and theories. Although computer-aided calculations are the basis for the design of composite materials nowadays, experimental testing is still required to confirm the simulations.

If the mechanical behaviour of a composite structure has to be simulated by using numerical models, but the exact input data, such as the ply structure or the material properties of the constituents, are not accurately known, the uncertainty in the simulation results is greatly increased. For this reason, optical measurement methods such as digital image correlation (DIC) are used to accurately determine the elastic constants of the anisotropic composites in the last years [24,25,26].

The digital image correlation (DIC) method, a non-contact and full-field deformation measurement method, has recently proven to be an efficient technique for the study of the composite structures [26,27]. By using this non-contact measuring technique, strain fields in materials can be studied by using virtual strain gauges or virtual extensometers, and it allows so-called local strain mapping to be carried out, offering accuracy examination of the strain distribution developed in a material [28,29].

The randomly spraying technique for obtaining speckle patterns monitored by the DIC method is the best method according to two recently published works [30,31]. Pan et al. [30] concluded that the randomly spraying technique provides the best quality after comparative analysis of the quality of the speckle patterns by using analysis of the colour intensity for the speckle patterns obtained by three methods: randomly spraying with black and white paints; using of a black marker pen on a composite film; and polishing by using sandpaper on a metal surface.

In processing the photos using the DIC method, the imaged surface applied with a random speckle pattern is divided into small subsets and the deformations of the surface are computed via algorithms by tracking the displacement of the subsets for an image (photo) comparatively with the reference photo corresponding to the undeformed structure. As a result, a complete full-field displacement map of the studied surface is obtained, from which desired strain analysis can be post-processed [24,32].

Additionally to the mechanical characterization of the composite materials, the DIC method was also used by Guo et al. [21] to reveal the effects of the hybridization mechanism on the carbon-glass fibre reinforced polymer composite rod. The accuracy of the full-field strains monitored using DIC reveal that the mechanical failure of the rods characterized by inter-layer hybridization was dominant by debonding at the shell–core interface.

Although there has been a lot of research analysing the mechanical performance of carbon/epoxy composites and Kevlar/epoxy composites in the literature [5,33,34,35], a lack of information exists regarding some mechanical properties of carbon/aramid/epoxy hybrid composite. For example, there are no publications investigating the Poisson’s ratio, because the authors reported only the tensile strength and modulus of elasticity [36]. The most recent research aimed to analyse the impact and post-impact properties of the composite materials reinforced with carbon-aramid fabrics [12,13].

Thus, it is crucial to propose a reliable and efficient method to characterize the mechanical properties of the carbon-aramid hybrid composite using the DIC method, in particular a method for the determination of the Poisson’s ratio $\nu_{12}$ (transverse contraction coefficient) corresponding to the reinforcement plane of the composite—a key parameter in analytical and numerical models of laminated composites [37].

On the other hand, the scientific literature lacks papers which analyse the mechanical behaviour of the hybrid carbon-aramid composites by using the analytical and numerical models.

The main purpose of this paper is to simulate the distribution of the strains and stresses developed in a carbon-aramid/epoxy composite material in tensile and bending tests, considering the elastic constants evaluated by using DIC method. For this purpose, a laminated composite material based on epoxy resin reinforced with eight layers of hybrid carbon-aramid woven fabric was investigated firstly by means of mechanical tests. The main objectives of this research are the following: (i) determination of the mechanical characteristics including Poisson ratio; (ii) applying the analytical models to the moduli of elasticity corresponding to the fictitious orthotropic material which behaves similarly to the carbon-aramid composite investigated; (iii) simulation of the mechanical behaviour by using finite element analysis (FEA) in order to evaluate the equivalent moduli of elasticity of the laminated carbon-aramid composite; (iv) comparison of the results obtained by numerical simulation with the experimental results.

All tests were performed on two sets of specimens: one set contained specimens whose length was parallel to the warp direction of the reinforced carbon-aramid woven fabric, and the other set contained specimens whose length was parallel to the weft direction.

## 2. Materials and Methods

### 2.1. Materials

**Figure 1:**
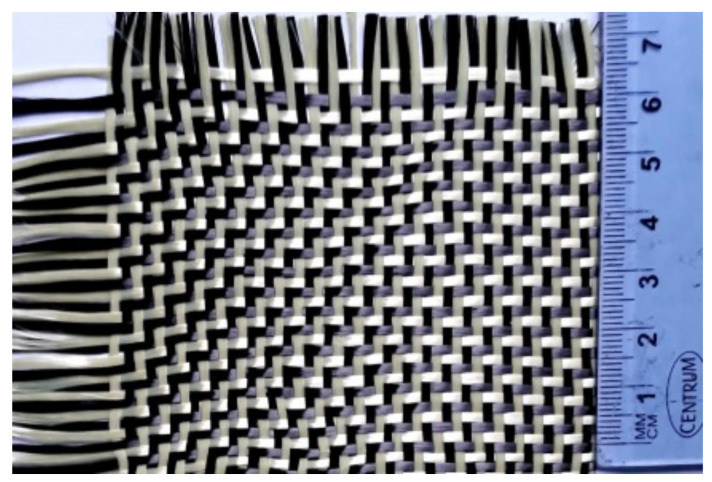
Photo of the SIGRATEX H W215-TW2/2 twill woven carbon-aramid fabric used to reinforce the laminated composite material tested (carbon fibres are black, aramid fibres are yellow).

Carbon-aramid woven fabric of type SIGRATEX H W215-TW2/2, manufactured by SGL Carbon (Wiesbaden, Germany), was used to reinforce the Epolam 2031 complex of epoxy resin and its corresponding Epolam 2031 hardener (Axson Technologies, Eaton Rapids, MI, USA). SIGRATEX H W215-TW2/2 carbon-aramid woven fabric, whose density is 215 g/m^2^, is a bidirectional twill hybrid fabric containing both kinds of fibres, carbon fibres and aramid fibres, on both directions (Figure 1) [38]. The thread count per centimetre was equal to 5.7 in both the warp and weft directions. The fineness of yarn was the same in both the warp and weft directions: 2000 dtex for the carbon fibres and 1600 dtex for the aramid fibres. Carbon fibre of type HT-3 kwas used to manufacture the SIGRATEX H W215-TW2/2 carbon-aramid woven fabric [38]. Carbon fibres of type 3 k meant that each tow contained 3000 strands (filaments) of carbon. HT carbon fibres were characterized by a low modulus of elasticity (less than 100 GPa) and high tensile strength (greater than 4.5 GPa) [39].

Epoxy resin of type Epolam 2031 was mixed with Epolam 2031 hardener before impregnation of the carbon-aramid woven fabric, with the mix ratio by volume being equal to 100:33 according to datasheet of the resin [40]. The density of the Epolam 2031 epoxy resin was 1.16 g/cm^3^, while the glass transition temperature was 120 °C. The density of Epolam 2031 hardener was 0.92 g/cm^3^. Epolam 2031 complex (epoxy resin and hardener) was characterized by a glass temperature of 138 °C and by the following mechanical characteristics: a tensile modulus of 3600 MPa; tensile strength of 80 MPa; flexural modulus of 2900 MPa; and flexural strength of 130 MPa [40].

The composite panel, whose dimensions were 600 mm × 460 mm, was made of eight layers reinforced with SIGRATEX H W215-TW2/2 twill carbon-aramid fabric. The fibre weight ratio was equal to 45 wt.%. The thickness of the composite panel was approximately equal with 2.6 mm. It has to be noted that the orientation of the reinforced carbon-aramid fabric was kept the same in all layers of the composite. The gel time of the mixture of Epolam 2031 epoxy resin and hardener was 110 min. according to the technical sheet of Epolam 2031 epoxy resin [40]. After moulding, the composite panel was kept at room temperature (20 °C ± 2 °C) for two weeks, before cutting of the specimens using jet cutting. Two sets of specimens corresponding to the warp and weft directions of the reinforcedt carbon-aramid woven fabric were cut for each type of mechanical test involved: the tensile test, the bending test using the three point method; and the impact test using the Charpy method.

### 2.2. Experimental Work Method

#### 2.2.1. Experimental Program in Tensile Test Combined with DIC Method

The universal testing machine LFV50-HM, 980 (Walter&Bai, Löhningen, Switzerland), whose maximum force was 50 kN, was used in tensile test. This machine had digital controls and the following data were recorded by its controlling software installed on a computer: tensile force *F*, elongation Δl of the tensile specimen and time *t*. According to the standard EN ISO 527-4 [41], the speed of loading was 1 mm/min. The data acquisition frequency was set to every second in controlling software of the machine prior to the tensile test.

**Figure 2:**
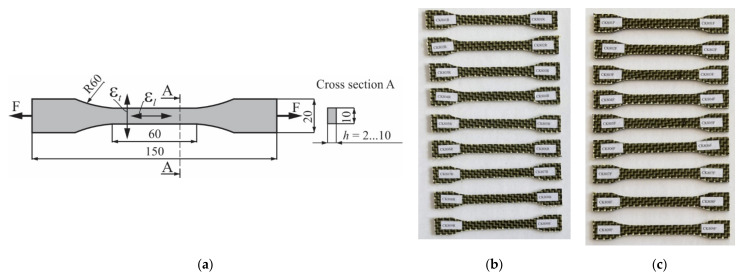
Tensile specimens: (**a**) dimensions of shape of the tensile specimens according to EN ISO 527-4 ($\varepsilon_{l}$—longitudinal strain, $\varepsilon_{t}$—transverse strain, all dimensions are expressed in mm), (**b**) tensile specimens whose length was parallel to the warp direction of the carbon-aramid fabric, and (**c**) tensile specimens whose length was parallel to the weft direction of the carbon-aramid fabric (the last letter R or F of the specimen code shows warp or weft direction, respectively).

The tensile specimens (Figure 2) were obtained by jet cutting. The dimensions and shape of the specimens (Figure 2a) were in accordance with the standard EN ISO 527-4 [41] and with the methodology used in work [26]. The tensile specimens whose length was parallel either to the warp direction or weft direction of the carbon-aramid reinforced fabric, are shown in Figure 2b or Figure 2c, respectively.

The aim of the tensile test was to determine the following properties of the composite material involved in this research: tensile modulus of elasticity, tensile strength, and Poisson’s ratio (transverse contraction coefficient). For this purpose, the tensile test was combined with the DIC method in order to use virtual extensometers for the determination of the elongation and strains of the specimens. Using the DIC method, the extensometer or strain gauges as measurement instruments were replaced with some virtual ones without additional costs for consumable materials (strain gauges) or for instruments (extensometer). This is just one of the advantages of the DIC method.

The DIC method is a digital optical method used in this research to obtain very accuracy displacement and strain fields of the entire tensile specimens at different times of tensile loading. The DIC method is based on tracking the position of the same pixel in some consecutively speckle images of the tensile specimens recorded during tensile test. The displacement and strain fields are obtained by using special digital virtual image correlation software dedicated to experimental mechanics. Practically, the current position of each pixel is tracked and compared with its initial position recorded in the first speckle image that corresponds to the unloaded tensile specimen. The quality of the random speckle pattern applied on the specimen ensures the accuracy of the results obtained using the DIC method [32].

**Figure 3:**
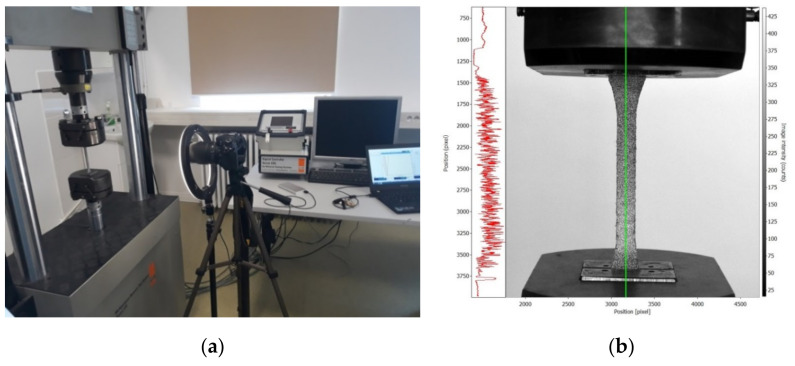
Tensile test combined with the DIC method: (**a**) experimental setup and (**b**) intensity gradient scale of the speckle pattern for the tensile specimen.

Figure 3 shows some photos from the experimental program carried out in the tensile test. The experimental setup of the tensile test combined with DIC method is shown in Figure 3a. Before testing, the tensile specimens were randomly sprayed consecutively with white paint and black paint in order to obtain the speckle patterns required for using the DIC method (Figure 3b). The spraying technique was chosen to obtain the speckle pattern because this method assures the best quality according to [30,31]. To maintain approximately the same size and quality of the speckle patterns, the spraying of the specimens was made by the same operator, at the same time, by using the same sprays with paints.

In this research, to evaluate the quality of the speckle patterns, we analysed the distribution of the colour intensity (Figure 3b) in the vertical direction of the speckle pattern used to monitor the full strain field of each tensile specimen monitored using the DIC method. The graph shown to the right side of Figure 3b shows the uniformity of the speckle pattern at the pixel-grey distribution scale level. The analysis of the distribution of the intensity colour of the speckle patterns provided the conclusion of a good quality of the speckle patterns in the case of all tensile specimens monitored by the DIC method.

The photo camera Nikon D7200 was used to record the speckle images of the tensile specimens during the tensile test. The acquisition frequency of the photos was set to one frame per second. The resolution of the photos acquired was set to 6000 × 4000 pixels and the camera lens AF-S NIKKOR18-105 mm f/3.5–5.6 ED VR was used.

An LED ring light source with 120 leds which may emit white, warm and neutral light at adjustable intensity using the wired remote control was used. The light source was connected by USB connection to the power source. The LED ring light source was set to white light at maximum intensity.

The time was the parameter used to correlate the data acquired by the tensile machine regarding the tensile force and the data recorded by the DIC method concerning the longitudinal and transverse strains, as long as the acquisition frequency was set to one second.

To measure the longitudinal strain $\varepsilon_{l}$ and transverse strains $\varepsilon_{t}$ of the tensile specimen at every second, the virtual gauge and virtual extensometers were considered as the region of interest in the DIC method. The main parameters set for the DIC method were that the subset size was 31 pixels, and the calculation step was 10 pixels.

The tensile modulus *E* of the laminated composite material reinforced with carbon-aramid fabric was computed as the slope of the linear portion of the curve of the normal stress σ related to the longitudinal strain $\varepsilon_{l}$. The Poisson’s ratio $\nu_{12}$ of the composite material in the reinforcement plane 12 was determined as the slope of the linear portion of the curve that plotted the variation of the transverse strains $\varepsilon_{t}$ as a function of the longitudinal strain $\varepsilon_{l}$. The linear portions considered for the both $\sigma -\varepsilon_{l}$ and $\varepsilon_{t}-\varepsilon_{l}$ curves corresponded to a variation of the longitudinal strain $\varepsilon_{l}$ between 0.002 and 0.007.

#### 2.2.2. Bending Test

The universal testing machine manufactured by Walter&Bai (Switzerland), whose maximum force is 100 kN, was used in the bending test. The work domain of the force actuator was 0–100 kN and the accuracy was $10^{-14}$ N. The force actuator was digitally controlled by the software DION7 (Walter&Bai, Löhningen, Switzerland). The three point method was used and the speed of loading was 1.5 mm/min according to the standard EN ISO 14125 [42]. The acquisition frequency was set to every 0.1 s in software which controlled the testing machine and the following data were recorded: time; bending force *F* and deflection *v* at the midpoint of the flexural specimen. The average flexural modulus of elasticity and the average flexural strength for each set of specimens corresponding to both the warp and weft directions of the reinforcement carbon-aramid woven fabric were determined. The flexural modulus of elasticity *E’* was computed by using the initial linear portion of the curves which plotted the variation of the bending force in function of the deflection *v*. The linear portion of the force–displacement (*F*-*v*) curve, established in accordance with standard EN ISO 14125 [42], was approximated with a linear function whose slope was ΔF/Δv. Then, the flexural modulus of elasticity *E′* was computed by using Equation (1) for each specimen:

(1) $$E^{\prime }=\left(l^{3}/48I_{z}\right)\left(\Delta F/\Delta v\right),$$

where *l* is the span of the flexural specimen between the simple supports, and $I_{z}$ represents the axial moment of inertia of the cross section of the specimen with respect to the neutral axis.

#### 2.2.3. Impact Test

**Figure 4:**
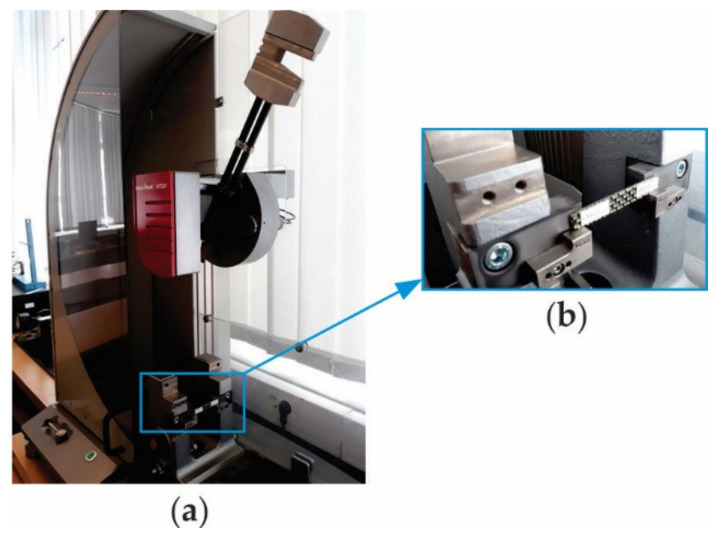
Charpy impact test setup: (**a**) pendulum impact tester HIT50P, and (**b**) photo of the Charpy specimen simply supported at its both ends.

For the Charpy impact test, we used the pendulum impact tester HIT50P (Zwick/Roell, Ulm, Germany) (Figure 4), whose maximum impact energy is equal to 50 J. The specimen was simply supported at both ends and the impact hammer struck the middle of the specimen in the impact test by the Charpy method.

The Charpy specimens with a rectangular shape whose dimensions were 80 mm × 10 mm were cut using a jet from the carbon-aramid composite plate according to the standard ISO 179-1 [43]. Prior to the impact test, the dimensions of the cross-section of each specimen were measured and noted.

The pendulum impact tester HIT50P with digital controlling measured and displayed the failure energy *W* for each specimen tested. The impact strength *K* or resilience corresponding to each specimen was computed as the ratio between the failure energy *W* and the area *A* of the specimen cross-section by using Equation (2):

(2) K=W/A.

### 2.3. Aspects Concerning the Analytical Models for Laminated Composite

In this section, some aspects are presented regarding the analytical models given in the scientific literature and used in this research in order to compute the elastic moduli of elasticity of the fictitious orthotropic material that is equivalent to the laminated hybrid carbon-aramid composite involved, in terms of mechanical behaviour in tensile and bending tests. The goal of this section is to compare the equivalent elastic moduli of the fictitious orthotropic material, computed by using the analytical model, with the values obtained by the tensile and bending tests. For this purpose, the main objective is to show the parameters which must be computed for supporting the calculus of the equivalent moduli of elasticity of the laminated hybrid carbon-aramid composite. The intermediary calculus relations which must be used to complete the calculus based on the elastic properties of each layer are also shown.

In the mechanics of composite materials, two coordinate systems are in operation: a local coordinate system, whose axes 1, 2 are aligned with the reinforcement directions of the layer, which correspond with the two yarn directions of the bidirectional carbon-aramid fabric involved in this research; and a global coordinate system xOyz that is arbitrarily chosen by the designer. The reduced stiffness matrix ${\left[\overset{𝄗}{Q}\right]}_{k}$ links the reduced stress vector ${\left\{\begin{array}{ccc} \sigma_{x} & \sigma_{y} & \tau_{xy} \end{array}\right\}}^{T}$ computed at the level of the arbitrary point located in the layer *k* and the reduced strain vector ${\left\{\begin{array}{ccc} \varepsilon_{x} & \varepsilon_{y} & \gamma_{xy} \end{array}\right\}}^{T}$ computed for the same point, with respect to the global coordinate system xOyz. Because the local coordinate system coincides with the global coordinate system in this research (all layers have the same orientation), the reduced stiffness matrix ${\left[\overset{𝄗}{Q}\right]}_{k}$ of any layer $k\;\left(k=\bar{1,N}\right)$, *N* being total number of layers, with respect to the global coordinate system is equal to the reduced stiffness matrix ${\left[Q\right]}_{k}$ related to the local coordinate system and it is computed by using Equation (3) in case of the thin composite layer, called the lamina [44]:

(3) $${\left[\overset{𝄗}{Q}\right]}_{k}={\left[Q\right]}_{k}=\left[\begin{array}{ccc} Q_{11} & Q_{12} & 0\\ Q_{12} & Q_{22} & 0\\ 0 & 0 & Q_{66} \end{array}\right]=\left[\begin{array}{ccc} E_{1}/\Delta & \nu_{12}E_{2}/\Delta & 0\\ \nu_{12}E_{2}/\Delta & E_{2}/\Delta & 0\\ 0 & 0 & G_{12} \end{array}\right],$$

where $\Delta =1-\nu_{12}\nu_{21}=1-\nu_{12}^{2}E_{2}/E_{1}$; $E_{1},\;E_{2}$ are the moduli of elasticity of the layer with respect to the reinforcing directions 1 and 2, respectively, corresponding to the yarn directions of the bidirectional reinforcement fabric; $G_{12}$ is the shear modulus of the layer with respect to the reinforcement plane 12; and $\nu_{12}$ is Poisson’s ratio of the layer with respect to the reinforcement plane 12.

The relation between the internal forces ${\left\{\begin{array}{ccc} \begin{array}{cc} N_{x} & N_{y} \end{array} & \begin{array}{cc} N_{xy} & M_{x} \end{array} & \begin{array}{cc} M_{y} & M_{xy} \end{array} \end{array}\right\}}^{T}$ developed at the level of the median surface of the laminated composite plate element, and strains and curvatures is given by Equation (4) constitutively [44]:

(4) $$\begin{array}{c} {\left\{\begin{array}{ccc} \begin{array}{cc} N_{x} & N_{y} \end{array} & \begin{array}{cc} N_{xy} & M_{x} \end{array} & \begin{array}{cc} M_{y} & M_{xy} \end{array} \end{array}\right\}}^{T}\\ \;\;\;\;\;\;\;=\left[\begin{array}{cc} \left[A\right] & \left[B\right]\\ \left[B\right] & \left[D\right] \end{array}\right]{\left\{\begin{array}{ccc} \begin{array}{cc} \varepsilon_{x}^{0} & \varepsilon_{y}^{0} \end{array} & \begin{array}{cc} \gamma_{xy}^{0} & k_{x}^{0} \end{array} & \begin{array}{cc} k_{y}^{0} & k_{xy}^{0} \end{array} \end{array}\right\}}^{T}, \end{array}$$

where $\varepsilon_{x}^{0},\;\varepsilon_{y}^{0}$ are the normal strains and $\gamma_{xy}^{0}$ is the shearing strain with respect to the median surface; and $k_{x}^{0},k_{y}^{0},k_{xy}^{0}$ are the curvatures. The terms of the stiffness matrix in plane [*A*], of the bending-stretching coupling matrix [*B*] and of the stiffness matrix in bending [*D*] are computed with Equation (5) [44]:

(5) $$\left\{\begin{array}{ll} A_{ij}=\overset{N}{\underset{k=1}{\sum }}{\left({\bar{Q}}_{ij}\right)}_{k}\left(z_{k}-z_{k-1}\right)=\overset{N}{\underset{k=1}{\sum }}{\left({\bar{Q}}_{ij}\right)}_{k}t_{k}; & D_{ij}=\frac{1}{3}\overset{N}{\underset{k=1}{\sum }}{\left({\bar{Q}}_{ij}\right)}_{k}{\left(z_{k}-z_{k-1}\right)}^{3}.\\ B_{ij}=\frac{1}{2}\overset{n}{\underset{k=1}{\sum }}{\left({\bar{Q}}_{ij}\right)}_{k}{\left(z_{k}-z_{k-1}\right)}^{2}; & ⬚ \end{array}\right.$$

Using Equation (4), we obtain Equation (6) that computes strain components and curvatures related to the internal forces:

(6) $${\left\{\begin{array}{ccc} \begin{array}{cc} \varepsilon_{x}^{0} & \varepsilon_{y}^{0} \end{array} & \begin{array}{cc} \gamma_{xy}^{0} & k_{x}^{0} \end{array} & \begin{array}{cc} k_{y}^{0} & k_{xy}^{0} \end{array} \end{array}\right\}}^{T}=\left[\begin{array}{cc} \left[\alpha \right] & \left[\beta \right]\\ \left[\beta \right] & \left[\delta \right] \end{array}\right]{\left\{\begin{array}{ccc} \begin{array}{cc} N_{x} & N_{y} \end{array} & \begin{array}{cc} N_{xy} & M_{x} \end{array} & \begin{array}{cc} M_{y} & M_{xy} \end{array} \end{array}\right\}}^{T}.$$

The laminated composite material involved in this research is made of layers reinforced with bidirectional carbon-aramid fabric, having the same orientation. Therefore, the composite material involved is a symmetric special orthotropic material, and thus matrices $\left[B\right]$ and $\left[\beta \right]$ are null in Equations (4) and (6), respectively. It follows that Equation (6) may be separated into Equations (7) and (8):

(7) $$\left\{\begin{array}{c} \varepsilon_{x}^{0}\\ \varepsilon_{y}^{0}\\ \gamma_{xy}^{0} \end{array}\right\}=\left[\begin{array}{ccc} \alpha_{11} & \alpha_{12} & 0\\ \alpha_{12} & \alpha_{22} & 0\\ 0 & 0 & \alpha_{66} \end{array}\right]\left\{\begin{array}{c} N_{x}\\ N_{y}\\ N_{xy} \end{array}\right\};$$

(8) $$\left\{\begin{array}{c} k_{x}^{0}\\ k_{y}^{0}\\ k_{xy}^{0} \end{array}\right\}=\left[\begin{array}{ccc} \delta_{11} & \delta_{12} & 0\\ \delta_{12} & \delta_{22} & 0\\ 0 & 0 & \delta_{66} \end{array}\right]\left\{\begin{array}{c} M_{x}\\ M_{y}\\ M_{xy} \end{array}\right\}.$$

#### 2.3.1. Analytical Model of the Laminated Composite Loaded in Plane

The objective of this section is to give the expressions of the equivalent elastic characteristics $\left(E_{x},\;E_{y},\;G_{xy},\nu_{xy}\right)$ of the fictitious orthotropic material that is equivalent in terms of mechanical behaviour, with the symmetric special orthotropic laminated composite loaded just within the reinforcement plane by the axial forces $N_{x},\;N_{y}$ and shearing force $N_{xy}$. Since no bending or torsion moments develop, the vector of curvatures ${\left(\begin{array}{ccc} k_{x}^{0} & k_{y}^{0} & k_{xy}^{0} \end{array}\right)}^{T}$ is a null vector according to Equation (8). It follows that the strain vector in the reinforcement plane is computed with Equation (9):

(9) $${\left\{\begin{array}{ccc} \varepsilon_{x} & \varepsilon_{y} & \gamma_{xy} \end{array}\right\}}^{T}={\left\{\begin{array}{ccc} \varepsilon_{x}^{0} & \varepsilon_{y}^{0} & \gamma_{xy}^{0} \end{array}\right\}}^{T}+z{\left\{\begin{array}{ccc} k_{x}^{0} & k_{y}^{0} & k_{xy}^{0} \end{array}\right\}}^{T}={\left\{\begin{array}{ccc} \varepsilon_{x}^{0} & \varepsilon_{y}^{0} & \gamma_{xy}^{0} \end{array}\right\}}^{T}.$$

Assuming that the stresses $\left(\sigma_{x},\;\sigma_{y},\;\tau_{xy}\right)$ are uniformly distributed on the plate thickness in case of in-plane loading, the strain vector with respect to the median surface of the orthotropic plate is computed with Equation (10) depending on the elastic properties of the fictitious orthotropic material and stresses [44]:

(10) $$\begin{array}{l} \left\{\begin{array}{c} \varepsilon_{x}^{0}\\ \varepsilon_{y}^{0}\\ \gamma_{xy}^{0} \end{array}\right\}=\left[\begin{array}{ccc} 1/E_{x} & -\nu_{xy}/E_{x} & 0\\ -\nu_{xy}/E_{x} & 1/E_{y} & 0\\ 0 & 0 & 1/G_{xy} \end{array}\right]\left\{\begin{array}{c} \sigma_{x}\\ \sigma_{y}\\ \tau_{xy} \end{array}\right\}\\ \;\;\;\;\;\;\;=\left[\begin{array}{ccc} 1/E_{x} & -\nu_{xy}/E_{x} & 0\\ -\nu_{xy}/E_{x} & 1/E_{y} & 0\\ 0 & 0 & 1/G_{xy} \end{array}\right]\frac{1}{t}\left\{\begin{array}{c} N_{x}\\ N_{y}\\ N_{xy} \end{array}\right\}. \end{array}$$

Equating the terms of Equations (7) and (10), the elastic properties $\left(E_{x},\;E_{y},\;G_{xy},\;\nu_{xy}\right)$ of the fictitious orthotropic material which represent the equivalent elastic properties of the symmetric special orthotropic laminated composite material loaded just in the reinforcement plane are computed with Equation (11) [44]:

(11) $$\left\{\begin{array}{cc} E_{x}=1/\left(t\alpha_{11}\right)=\left(A_{11}A_{22}-A_{12}^{2}\right)/\left(tA_{22}\right); & G_{xy}=1/\left(t\alpha_{66}\right)=A_{66}/t;\\ E_{y}=1/\left(t\alpha_{22}\right)=\left(A_{11}A_{22}-A_{12}^{2}\right)/\left(tA_{11}\right); & \nu_{xy}=-t\alpha_{12}E_{x}=-\alpha_{12}/\alpha_{11}=A_{12}/A_{22}. \end{array}\right.$$

#### 2.3.2. Analytical Model of the Laminated Composite Subjected to Bending

The objective of this section is to give the expressions of the equivalent elastic characteristics $\left(E_{x}^{\prime },\;E_{y}^{\prime },\;G_{xy}^{\prime },\;\nu_{xy}^{\prime }\right)$ of the fictitious orthotropic material which behaves similarly to the symmetric special orthotropic laminated composite loaded just by the bending moments $\left(M_{x},\;M_{y}\right)$ and by the torsion moment $M_{xy\;}$. Since no force develops within the reinforcement plane, the strain vector with respect to the median surface is a null vector according to Equation (7). Thus, the strain vector in the reinforcement plane is computed with Equation (12):

(12) $${\left\{\begin{array}{ccc} \varepsilon_{x} & \varepsilon_{y} & \gamma_{xy} \end{array}\right\}}^{T}={\left\{\begin{array}{ccc} \varepsilon_{x}^{0} & \varepsilon_{y}^{0} & \gamma_{xy}^{0} \end{array}\right\}}^{T}+z{\left\{\begin{array}{ccc} k_{x}^{0} & k_{y}^{0} & k_{xy}^{0} \end{array}\right\}}^{T}=z{\left\{\begin{array}{ccc} k_{x}^{0} & k_{y}^{0} & k_{xy}^{0} \end{array}\right\}}^{T}.$$

Considering the relation between the vector of moments and reduced stress vector corresponding to the stress components developed within the plane *xy* of the fictitious orthotropic plate and then replacing the relations between stresses and strains, Equation (13) is obtained.

(13) $$\left\{\begin{array}{c} M_{x}\\ M_{y}\\ M_{xy} \end{array}\right\}=\int_{-t/2}^{t/2}\left\{\begin{array}{c} \sigma_{x}\\ \sigma_{y}\\ \tau_{xy} \end{array}\right\}zdz=\int_{-t/2}^{t/2}{\left[\begin{array}{ccc} 1/E_{x}^{\prime } & -\nu_{xy}/E_{x}^{\prime } & 0\\ -\nu_{xy}/E_{x}^{\prime } & 1/E_{y}^{\prime } & 0\\ 0 & 0 & 1/G_{xy}^{\prime } \end{array}\right]}^{-1}\left\{\begin{array}{c} \varepsilon_{x}\\ \varepsilon_{y}\\ \gamma_{xy} \end{array}\right\}zdz$$

Replacing the strain vector ${\left\{\begin{array}{ccc} \varepsilon_{x} & \varepsilon_{y} & \gamma_{xy} \end{array}\right\}}^{T}$ from Equation (12) in Equation (13), Equation (14) is obtained.

(14) $$\begin{array}{l} \left\{\begin{array}{c} M_{x}\\ M_{y}\\ M_{xy} \end{array}\right\}={\left[\begin{array}{ccc} 1/E\prime_{x} & -\nu_{xy}/E\prime_{x} & 0\\ -\nu_{xy}/E\prime_{x} & 1/E\prime_{y} & 0\\ 0 & 0 & 1/G\prime_{xy} \end{array}\right]}^{-1}\left\{\begin{array}{c} k_{x}^{0}\\ k_{y}^{0}\\ k_{xy}^{0} \end{array}\right\}\int_{-t/2}^{t/2}z^{2}dz\\ \;\;\;\;\;\;\;=\frac{t^{3}}{12}{\left[\begin{array}{ccc} 1/E\prime_{x} & -\nu_{xy}/E\prime_{x} & 0\\ -\nu_{xy}/E\prime_{x} & 1/E\prime_{y} & 0\\ 0 & 0 & 1/G\prime_{xy} \end{array}\right]}^{-1}\left\{\begin{array}{c} k_{x}^{0}\\ k_{y}^{0}\\ k_{xy}^{0} \end{array}\right\}. \end{array}$$

Using Equation (14), the vector of curvatures ${\left\{\begin{array}{ccc} k_{x}^{0} & k_{y}^{0} & k_{xy}^{0} \end{array}\right\}}^{T}$ is expressed in function of the vector of moments with Equation (15) [44]:

(15) $$\left\{\begin{array}{c} k_{x}^{0}\\ k_{y}^{0}\\ k_{xy}^{0} \end{array}\right\}=\frac{12}{t^{3}}\left[\begin{array}{ccc} 1/E\prime_{x} & -\nu_{xy}/E\prime_{x} & 0\\ -\nu_{xy}/E\prime_{x} & 1/E\prime_{y} & 0\\ 0 & 0 & 1/G\prime_{xy} \end{array}\right]\left\{\begin{array}{c} M_{x}\\ M_{y}\\ M_{xy} \end{array}\right\}.$$

Comparing Equations (8) and (15), the equivalent elastic properties $\left(E\prime_{x},\;E\prime_{y},\;G\prime_{xy},\;\nu \prime_{xy}\right)$ of the the symmetric special orthotropic laminated composite material loaded just by bending moments $\left(M_{x},\;M_{y}\right)$ and torsion moment $M_{xy}$ are computed by using the relations given in Equation (16) [44]:

(16) $$\left\{\begin{array}{cc} E\prime_{x}=12/\left(t^{3}\delta_{11}\right)=12\left(D_{11}D_{22}-D_{12}^{2}\right)/\left(t^{3}D_{22}\right); & G\prime_{xy}=12/\left(t^{3}\delta_{66}\right)=12D_{66}/t^{3};\\ E\prime_{y}=12/\left(t^{3}\delta_{22}\right)=12\left(D_{11}D_{22}-D_{12}^{2}\right)/\left(t^{3}D_{11}\right); & \nu_{\prime xy}=-t^{3}\delta_{12}E_{x}/12=-\frac{\delta_{12}}{\delta_{11}}=\frac{D_{12}}{D_{22}}. \end{array}\right.$$

### 2.4. Simulation Method for Mechanical Tests

Finite element analysis is used for simulation of the stress and strains state which develop in tensile and mechanical tests. One of the specific problems in the simulation of tensile and bending tests is that concerning the modelling of the laminated composite material involved in this research. The technique of modelling of the composite material through a discrete number of layers is used. The material assigned to each layer is defined by elastic properties that characterize an orthotropic material similar to the composite layer bidirectionally reinforced with carbon-aramid woven fabric. This means that macroscopic modelling was used for layers, since these layers were defined by using layer properties. The simulation methodology using finite element models was described separately for tensile and bending tests.

#### 2.4.1. Simulation of the Tensile Test

The numerical simulation of the tensile test of the specimen made of laminated composite material involved in this research was used by using the software Abaqus, student version. The major objective of this section is to evaluate the curve of the tensile force applied related to the elongation of a specimen portion located between two mark points in order to obtain the equivalent modulus of elasticity of the tensile specimen, obtained by means of FEA.

**Figure 5:**
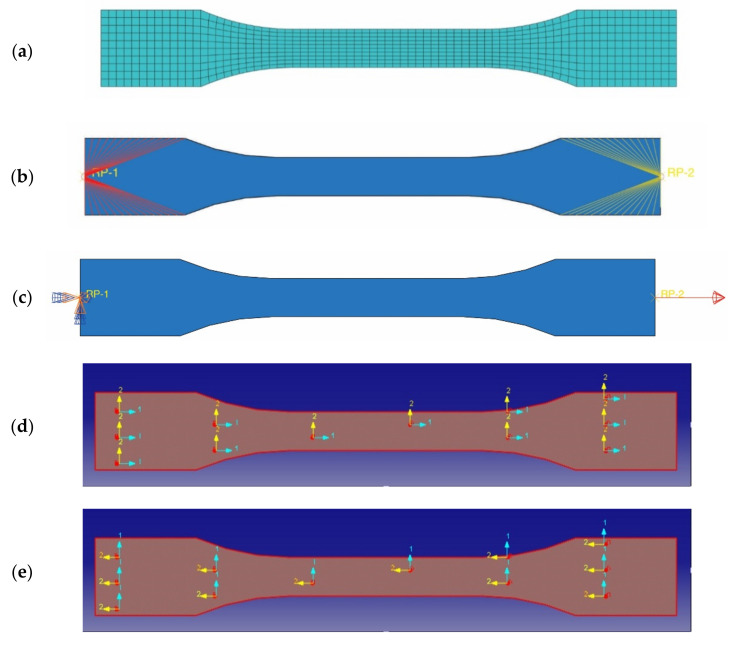
Numerical model for simulation of the tensile test: (**a**) finite element model, (**b**) coupling constraint, (**c**) defining of load and boundary condition, (**d**) material axis 1 is parallel to the specimen length, and (**e**) material axis 2 is parallel to the specimen length.

Firstly, the numerical model of the tensile specimen (Figure 5) was made by using S4R shell elements, which are linear finite elements with four nodes and are intended for thin or thick shells. The finite element model of the tensile specimen is shown in Figure 5a. Two coupling constraints controlled by two reference points (Figure 5b) were defined to simulate the fixing of the tensile specimen in the clamping jaws of the tensile machine and for applying of the tensile force to the other end of the bone shape specimen. Then, the tensile force of 10 kN was applied to the right control reference point in the numerical model, while the boundary conditions for the embedded end were applied to the second control reference point (Figure 5c).

Regarding the modelling of the laminated composite material which is assigned to the numerical model of the tensile specimen, the technique of modelling of the material through eight thin layers is used because the laminated composite involved is made of eight layers reinforced with carbon-aramid woven fabric. The material assigned to each layer is the same and it is defined as lamina type in Abaqus software, which is an orthotropic material. The elastic properties of each orthotropic layer are defined so that the modulus of elasticity assigned to the axis 1 is equal to the tensile modulus of elasticity determined for tensile specimens whose length is parallel to the warp direction of the reinforcement carbon-aramid woven fabric. The modulus of elasticity assigned to the material axis 2 is equal to the tensile modulus of elasticity determined in the tensile test applied in the weft direction of the reinforcement carbon-aramid woven fabric. In this context, for simulation of the tensile test applied in the warp direction, the material orientation was set like is shown in Figure 5c. To simulate the tensile test applied in weft direction, the material orientation was set in the manner shown in Figure 5d.

**Figure 6:**
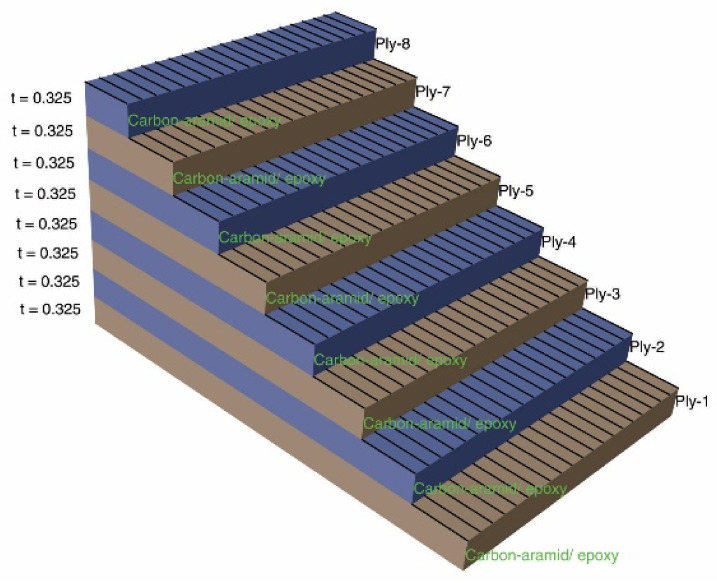
Layup defined for all layers of the hybrid laminated composite assigned to the model of the tensile specimen.

The composite layup assigned to the finite element model of the tensile specimen is presented in Figure 6. All layers had the same thickness of 0.325 mm, considering that the average thickness of the carbon-aramid/epoxy composite material was equal to 2.6 mm according to the measurements from the experimental program of this research. The orientation angle of the orthotropic material assigned to all layers was set to be equal with zero.

The stress–strain curve obtained by simulation of the tensile test was finally compared with the stress–strain curves experimentally obtained.

#### 2.4.2. Simulation of the Bending Test

**Figure 7:**
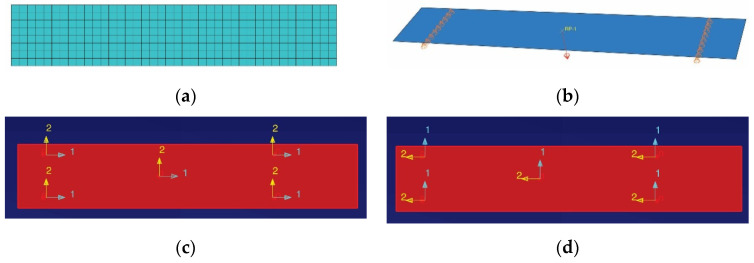
Numerical model for simulation of the bending test: (**a**) finite element model, (**b**) load and boundary conditions, (**c**) material axis 1 is parallel to the specimen length, and (**d**) material axis 2 is parallel to the specimen length.

In Figure 7, the numerical model used to simulate the stress and strain distributions in the bending test by using the three point method is presented. The finite element model contained 320 shell elements of type S4R and is shown in Figure 7a. The concentrated force of 80 N was applied to the reference point located at midpoint of the flexural specimen (Figure 7b), which controlled the middle of the specimens because a coupling constrain was defined. The load of 80 N corresponded to the upper limit of the linear portion of the force–displacement curve, for which the flexural modulus was computed.

The laminated composite material assigned to the flexural specimen was defined in the same manner as for the simulation of the tensile test. The flexural specimen also contained eight layers. Each layer had the thickness 0.325 mm and the orientation angle was set to zero, as in the case of the simulation of the tensile test.

The orientation angle of the orthotropic material assigned to all layers was set in the same manner as for the simulation of the tensile test: axis 1 of the material was parallel to the specimen length (Figure 7c) to simulate the flexural behaviour in the warp direction; axis 2 of the material was parallel to the specimen length (Figure 7d) to simulate the flexural behaviour in the weft direction.

The force–displacement curve was obtained by means of the simulation of the flexural test and by using its slope to compute the equivalent modulus of elasticity for the composite specimen.

## 3. Results

### 3.1. Experimental Results

#### 3.1.1. Tensile Properties

**Figure 8:**
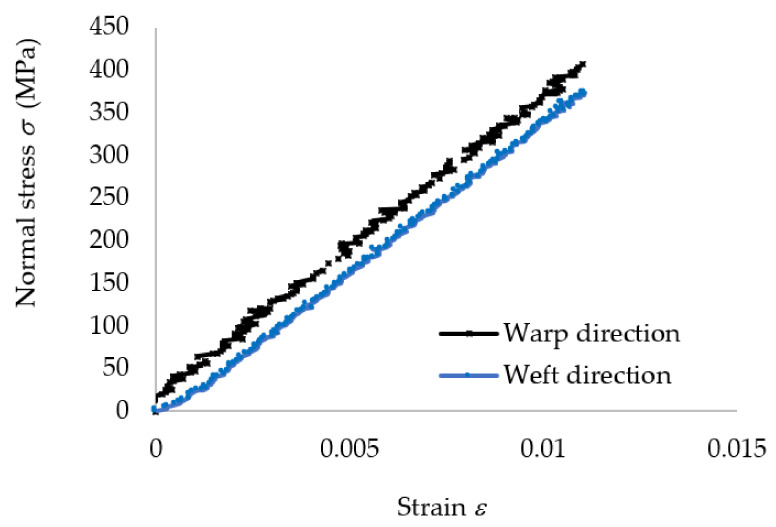
Mean stress–strain curves recorded in tensile tests on warp and weft direction of the carbon-aramid reinforcement fabric.

In Figure 8, the median stress–strain $\left(\sigma -\varepsilon \right)$ curves obtained in tensile tests applied to specimens whose length was parallel either to the warp direction or weft direction of the reinforcement carbon-aramid woven fabric are shown. It should be noted that the longitudinal strains used to plot the graph shown in Figure 8 were obtained by monitoring with DIC the full strain field by using virtual extensometer on longitudinal direction of the tensile specimen. Therefore, determination of the tensile moduli of the carbon-aramid composite material in both the warp and weft directions was based on DIC measurement.

We note that the slope of the stress–strain curve corresponding to the warp direction is greater than the slope of the curve corresponding to the weft direction (Figure 8). Thus, the composite material reinforced with carbon-aramid is a little stiffer in the warp direction than in the weft direction corresponding to the reinforced woven fabric.

The tensile properties obtained by testing all specimens are shown in Table 1.

**Figure 9:**
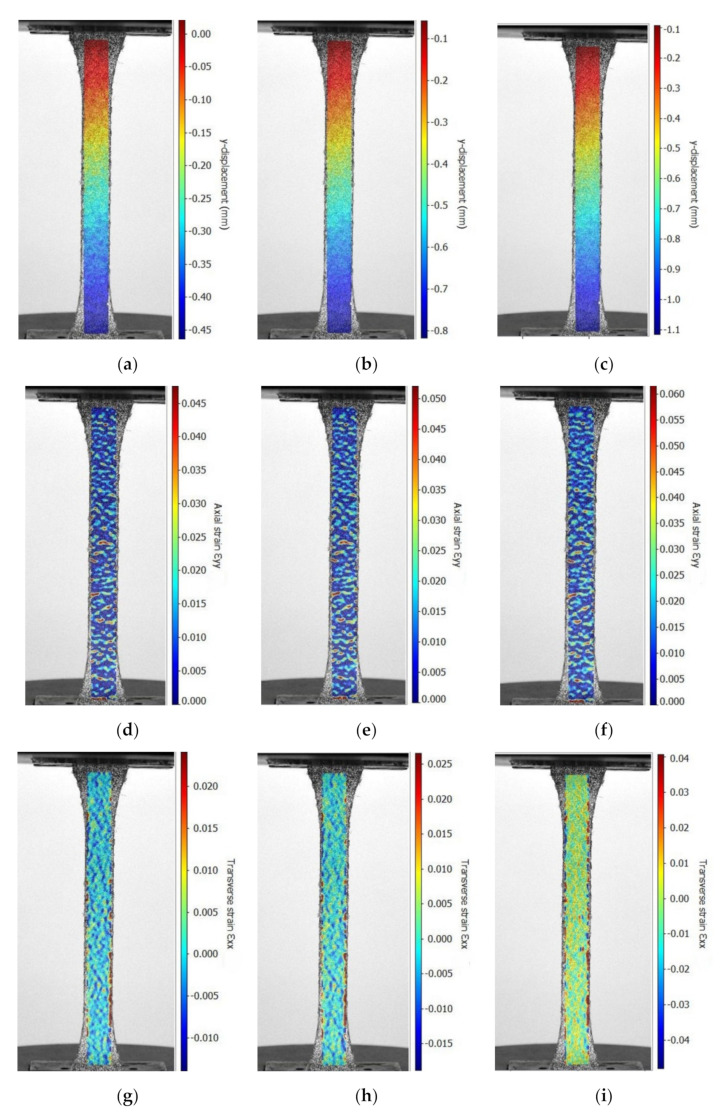
Results obtained by the DIC method in the tensile test applied in the warp direction corresponding to the carbon-aramid fabric: (**a**–**c**) displacement in longitudinal direction *y* of the tensile specimen, (**d**–**f**) longitudinal strain $\varepsilon_{l}$ (i.e., $\varepsilon_{yy}$), and (**g**–**h**) transverse strain $\varepsilon_{t}$ (i.e., $\varepsilon_{xx}$) (normal stress evolution: (**a**,**d**,**g**) 199.46 MPa; (**b**,**e**,**h**) 302.67 MPa; (**c**,**f**,**i**) 398.11 MPa).

The photos acquired every second during the tensile test were processed by using the DIC method. All results (displacement, strains) were computed for a rectangular area of interest defined for each specimen (Figure 9) in order to visualize their evolution in time, until the breaking of the specimen (for every second in this research). We obtained plots which show the distribution of the following quantities over the area of interest: displacement in the axial direction y of the tensile specimen (Figure 9a–c); longitudinal strain $\varepsilon_{l}$. in the direction of the tensile force (Figure 9d–f); and transverse strain $\varepsilon_{t}$ (Figure 9g–i). Herein, just some plots are given, which were acquired by the DIC method at different levels of loading corresponding to the following values of normal stress: 199.46 MPa (Figure 9a,d,g); 302.67 MPa (Figure 9b,e,h); 398.11 MPa (Figure 9c,f,i).

**Figure 10:**
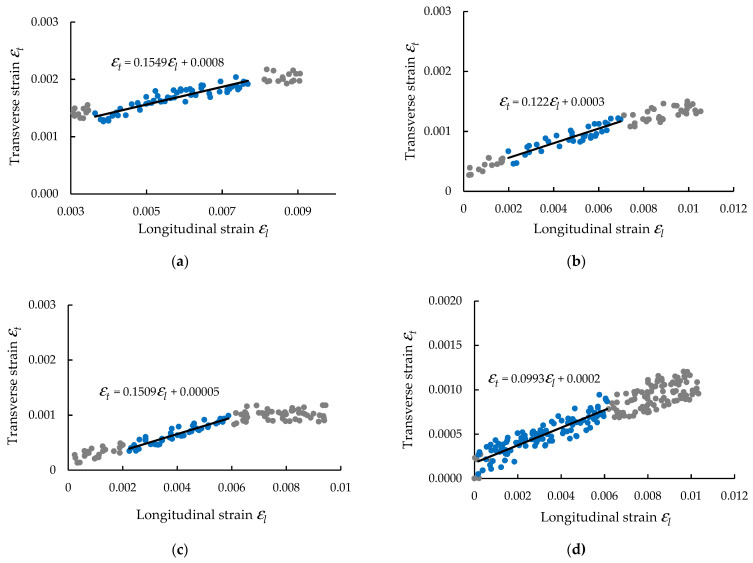

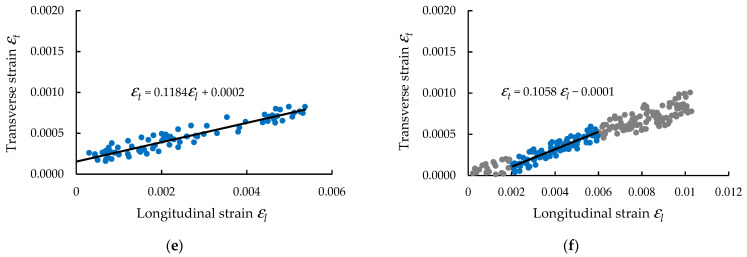
Curves of transverse strain $\varepsilon_{t}$ related to longitudinal strain $\varepsilon_{l}$ for determining the Poisson’s ratio ν_12_ by means of DIC method with respect to the both directions of carbon-aramid reinforced fabric: (**a**–**c**) warp direction, and (**d**–**f**) weft direction.

By defining three virtual extensometers on each direction of the tensile specimen (longitudinal and transverse directions), the data were defined using the DIC method regarding the history of the longitudinal strain $\varepsilon_{l}$ and transverse strain $\varepsilon_{t}$ during the tensile test for each specimen. In order to compute the Poisson ratio $\nu_{12}$ in the reinforcement plane with carbon-aramid woven fabric, the curve of the transverse strain $\varepsilon_{t}$ related to the longitudinal strain $\varepsilon_{l}$ was plotted for each specimen, as shown in Figure 10.

The data of the $\varepsilon_{t}-\varepsilon_{l}$ curve corresponding to the linear portion of the stress–strain curve (i.e., variation of the longitudinal strain between 0.002 and 0.007), were approximated with a linear function by using the method of the least squares (Figure 10). The slope of this linear function represents the Poisson ratio $\nu_{12}$ of the composite material in the reinforcement plane with carbon-aramid fabric. In Figure 10a–c, the $\varepsilon_{t}-\varepsilon_{l}$ curves and the approximation function recorded for tensile specimens whose length is parallel to the warp direction of the reinforcement carbon-aramid woven fabric are plotted. In the same manner, in Figure 10d–f, the results obtained for the tensile specimens whose length is parallel to the weft direction of the reinforcement carbon-aramid woven fabric are shown.

The large scattering of the results on the $\varepsilon_{t}-\varepsilon_{l}$ curves is mainly caused by the inhomogeneity of the composite layer because the reinforcing woven fabric is made of both carbon yarns and aramid fibres in both directions (Figure 1). According to the technical sheet of SIGRATEX H W215-TW2/2 carbon-aramid woven fabric [38], just 5.7 threads are distributed within 10 mm in both the warp and weft directions. Taking into account that the width is equal to 10 mm at the middle of the tensile specimen, a composite layer contains five or six threads and just half are of the same type (carbon threats or aramid threats). Furthermore, the tensile strength is greater than 4.5 GPa for the HT carbon fibres used to manufacture SIGRATEX H W215-TW2/2 carbon-aramid woven fabric [38,39], while the tensile strength of the aramid fibres is 2.4–3 GPa [45]. It follows that the aramid fibres break first, before the rupture of the carbon fibres in tensile tests of the composite material reinforced with hybrid carbon-aramid woven fabric. On the other hand, the HT carbon fibres are characterized by the tensile modulus of elasticity less than 100 GPa [39], that leads to normal strain $\varepsilon_{r}$ at failure greater than 0.045 (4.5%). Because the normal strain at rupture $\varepsilon_{r}$ for the aramid fibres is in the range 1.9–4.5% [45].

All results obtained for the Poisson’s ratio are shown in the last column of Table 1.

#### 3.1.2. Bending Properties

**Figure 11:**
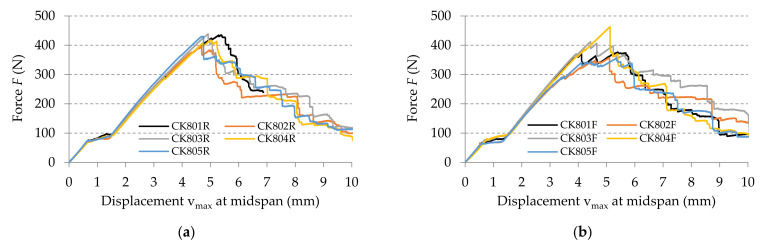
Force–displacement (*F*-*v*) curves recorded in bending tests, in the directions of the reinforcement fabric: (**a**) warp direction, and (**b**) weft direction.

The force–displacement (*F*-v) curves recorded in bending tests are plotted in Figure 11 for all specimens tested.

After processing of the experimental data according to the European standard EN-ISO 14125 [42], the flexural properties (flexural modulus of elasticity, flexural strength) are determined and the results are synthesized in Table 2. For each specimen, the flexural modulus of elasticity was computed by using Equation (1) for the experimental data corresponding to the linear portion of the force–displacement (*F*–*v*) curves, for variation of the maximum displacement *v*_max_ between 0.1 and 0.6 mm according to the European standard EN-ISO 14125 [42]. By using the method of least squares, this portion was approximated with a linear function whose slope ΔF/Δv was replaced in Equation (1).

#### 3.1.3. Impact Properties

The pendulum impact tester HIT50P recorded the failure energy *W* determined by Charpy impact testing for each specimen. Then, the impact strength *K* is computed by using Equation (2) for each specimen. The average values of the impact properties and their corresponding stdev values are shown in Table 3 for each set of impact specimens: one set of specimens whose length is parallel to the warp direction of the reinforcement carbon-aramid woven fabric, and another set of specimens whose length is parallel to the weft.

**Figure 12:**
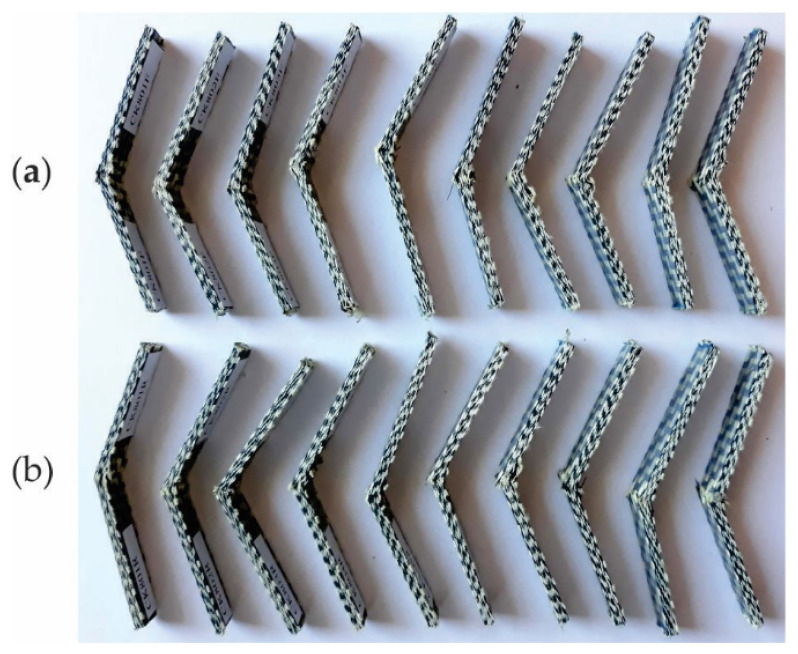
Specimens after Charpy impact testing: (**a**) impact specimens whose length is parallel to warp direction of carbon-aramid fabric, and (**b**) impact specimens whose length is parallel to weft direction of carbon-aramid fabric.

It is observed that the specimens are not completely broken in Charpy impact testing (Figure 12).

### 3.2. Results Obtained by Analytical Method

Taking into account the median thickness of 2.6 mm for the panel manufactured by epoxy resin reinforced with eight layers of carbon-aramid woven fabric, the thickness of one layer is considered to be equal to 0.325 mm in analytical model of the composite material.

Considering the elastic properties obtained in tensile testing of the composite material involved in this research, the terms of the stiffness matrix $\left[Q\right]$ of the composite layer are computed by using Equation (3) both for the case when the length of the flexural specimen is parallel to the warp direction of the reinforcement woven fabric and for the case when the length of the flexural specimen is parallel to the weft direction. The results are shown in Table 4.

In Table 5, the terms of the stiffness matrix $\left[Q\prime \right]$ of the composite layer are shown, computed by Equation (3) considering the elastic properties obtained by bending tests.

Using Equation (5), the components of the two stiffness matrices [*A*] and [*D*] are computed and the results are shown in Table 6. Using the first relation from Equation (11), we computed the tensile modulus of elasticity E corresponding to the fictitious orthotropic material that is equivalent to the composite material investigated in this research, in terms of the mechanical traction behaviour (see the penultimate column of Table 6).

Using the first relation from Equation (16), we computed the flexural modulus of elasticity *E′* corresponding to the fictitious orthotropic material that is equivalent to the composite material investigated, in terms of the mechanical behaviour in bending tests (see the last column of Table 6).

### 3.3. Results Obtained by Means of FEA

Since all orthotropic layers of the numerical model of the tensile specimen are characterized by the same elastic properties and have the same orientation angle, the distributions of the both normal stress and normal strain over the specimen thickness are constant in the case of the tensile test. In this context, it is not required to show the distributions of stresses and strains for each layer of the tensile specimen whose simulation results are analysed.

**Figure 13:**
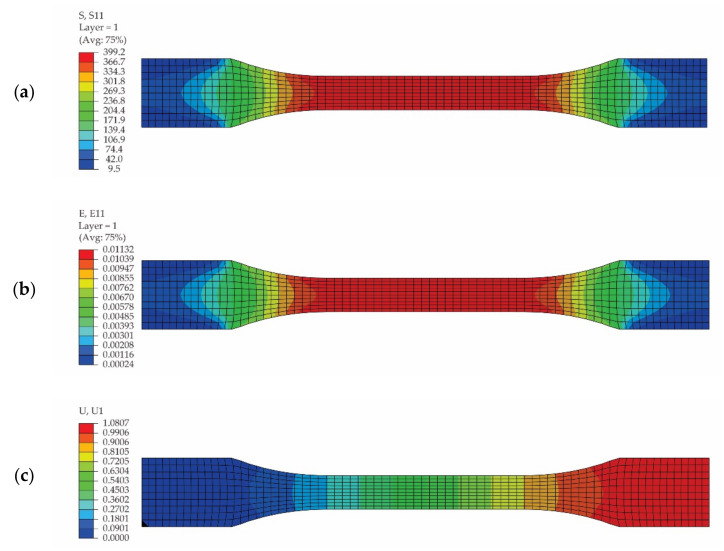
Results obtained by simulation of the tensile test of the specimen whose length is parallel to the warp direction of the reinforcement carbon-aramid fabric: (**a**) distribution of the normal stresses (denoted as S11), (**b**) distribution of the normal strains on longitudinal direction of specimen (denoted as E11), and (**c**) displacements on the specimen direction (denoted as U1).

In Figure 13, the results obtained by using the numerical model of the tensile specimen loaded on warp direction of the reinforcement carbon-aramid fabric are plotted. The distributions of the normal stresses and of the normal strains in the direction of the specimen are shown in Figure 13a and Figure 13b, respectively. The plot of the displacement on the direction of the specimen is shown in Figure 13c.

Analysing the results shown in Figure 13 obtained for the tensile specimen loaded on warp direction of the reinforcement carbon-aramid fabric, we may note the following: the maximum normal stress is 399.2 MPa; the maximum normal strain is 0.01132; and maximum displacement is 1.081 mm.

**Figure 14:**
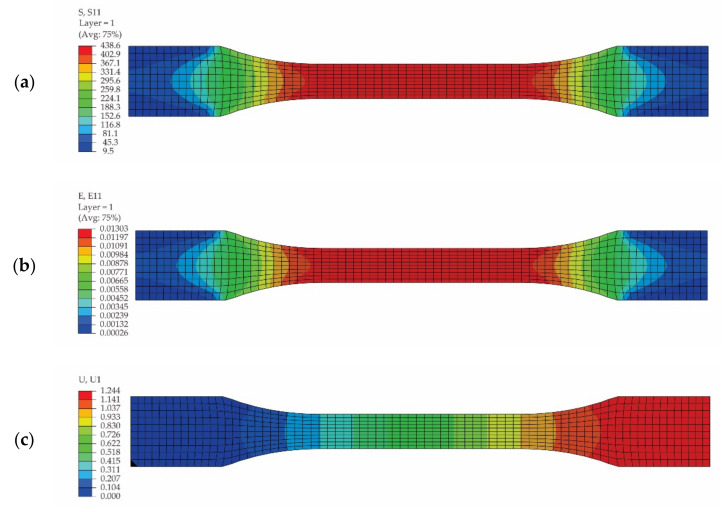
Results obtained by simulation of the tensile test of the specimen whose length is parallel to the weft direction of the carbon-aramid fabric: (**a**) distribution of the normal stresses (denoted as S11), (**b**) distribution of the normal strains in the longitudinal direction of specimen (denoted as E11), and (**c**) distribution of the displacements in the specimen direction (denoted as U1).

Figure 14 shows the results obtained by means of FEA for the tensile specimen loaded in the weft direction of the reinforcement carbon-aramid fabric: the distribution of the normal stresses in the direction of the specimen (Figure 14a); the distribution of the normal strains (Figure 14b); and the displacement in the direction of the specimen (Figure 14c). The following results were recorded through the simulation of the tensile specimen loaded in the weft direction of the reinforcement carbon-aramid fabric: the maximum normal stress is 438.6 MPa; the maximum normal strain is 0.01303; and the maximum displacement is 1.244 mm.

It may be observed that the maximum normal stress for the specimen loaded in the weft direction is 12.14% less than the maximum normal stress corresponding to the specimen loaded in the warp direction.

**Figure 15:**
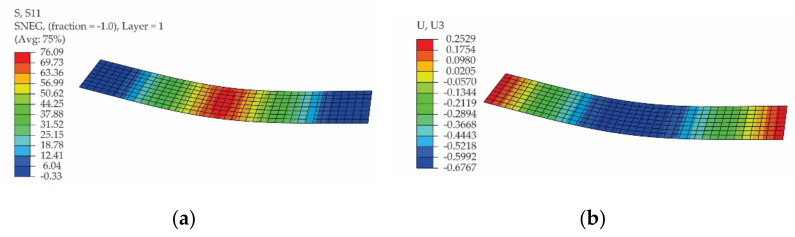
Results obtained by simulation of the bending test of the specimen whose length is parallel to the warp direction of the carbon-aramid fabric: (**a**) normal stress in the specimen direction (denoted as S11), and (**b**) vertical displacement (denoted as U3).

**Figure 16:**
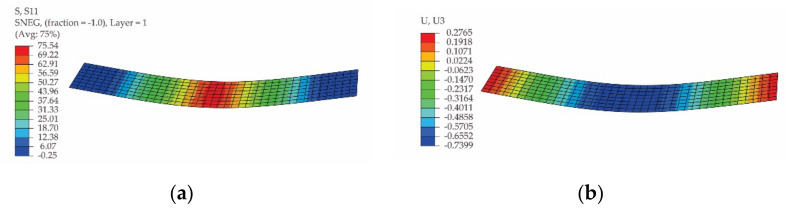
Results obtained by simulation of the bending test of the specimen whose length is parallel to the weft direction of the carbon-aramid fabric: (**a**) normal stress on specimen direction (denoted S11), and (**b**) vertical displacement (denoted U3).

In Figure 15 and Figure 16, the results obtained by simulation of the bending test for the specimen whose length is parallel to the warp direction and for the specimen whose length is parallel to the weft direction of the carbon-aramid woven fabric are presented, respectively. Analysing the results shown in Figure 15b and Figure 16b, it may be remarked that the maximum displacement of 0.2529 mm (Figure 15b) recorded for the flexural specimen whose length is parallel to the warp direction of the carbon-aramid fabric is 8.53% less than the maximum displacement of 0.2765 mm (Figure 16b) obtained for the specimen whose length is parallel to the weft direction of the fabric.

### 3.4. Experimental versus Theoretical Results

Analysing the results plotted in Figure 13a,b, for simulation of the tensile test for the specimen whose length is parallel to the warp direction of the reinforcement carbon-aramid fabric, it may be observed that the maximum normal strain $\varepsilon_{l}$ of 0.01132 in the longitudinal direction of specimen corresponds to the maximum normal stress of 399.2 MPa. In Table 7, a comparison between the results obtained using the DIC method and the results obtained by means of FEA concerning the longitudinal strains $\varepsilon_{l}$. corresponding to different values of the normal stresses in the case of one tensile specimen whose length is parallel to the warp direction of the reinforcement carbon-aramid fabric is shown. The normal stresses are not integer values in Table 7 because the longitudinal strains were extracted by the DIC method for different moments of time, and then these values were correlated with the forces and with the normal stresses recorded by the tensile machine.

On the other hand, the normal stress of 398.11 MPa corresponds with the longitudinal stress of 0.10752 (Table 7), as monitored by the DIC method. The full field of the longitudinal strains of the hybrid composite monitored using DIC, shown in Figure 9f for the tensile force of 11.162 kN that corresponds to the normal stress of 398.11 MPa, shows that dark blue to light blue predominates on plotting the colour contour. The mean value of the blue colour contour is about 0.010 for the longitudinal strain in Figure 9f.

The errors of the results obtained using the DIC method with respect to the results obtained by means of FEA are computed in the last column of Table 7. The errors are acceptable because the longitudinal strains obtained by means of FEA were computed by using the average values of the tensile properties corresponding to the entire set of tensile specimens, while the longitudinal strains obtained using the DIC method correspond just to one tensile specimen. For this reason, it can be considered that it is much better to compare the stress–strain $\left(\sigma -\varepsilon \right)$ curves obtained by means of FEA with the experimental curves based on DIC measurements.

The history output data concerning the displacements of the two nodes located on the middle portion of the tensile specimen, with a constant cross section, were reported for some increments. Thus, we obtained the curve of the tensile force related to the elongation of the portion located between the mark nodes. Finally, the curve of stress related to strain is obtained, and its slope is the modulus of elasticity obtained by means of FEA.

**Figure 17:**
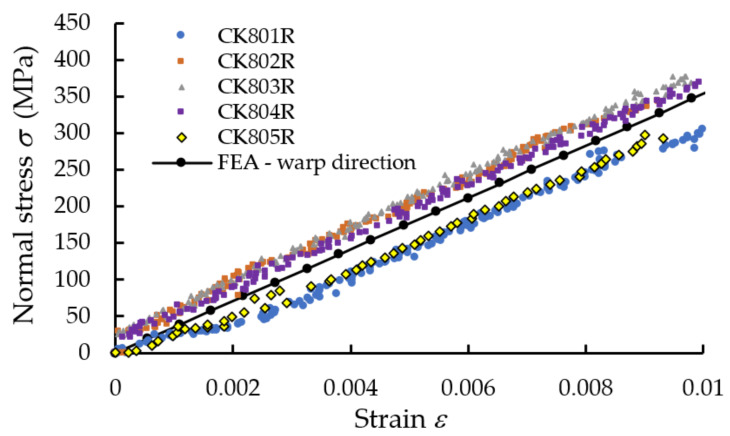
Comparison between stress–strains curves obtained by tensile test applied in the warp direction of the carbon-aramid reinforcement fabric and the curve obtained by numerical simulation.

**Figure 18:**
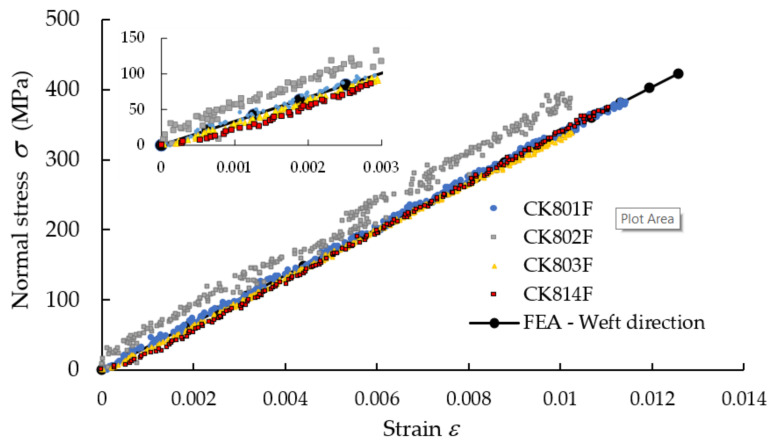
Comparison between stress–strains curves obtained by tensile test applied in the weft direction of the carbon-aramid reinforcement fabric and the curve obtained by numerical simulation.

In Figure 17, the stress–strain curve obtained by means of FEA is superimposed over the experimental data in the case of the tensile specimens whose length is parallel to the warp direction of the carbon-aramid reinforcement fabric. In the same manner, Figure 18 comparatively shows the stress–strain curve obtained by means of FEA and the experimental stress–strain curves recorded for the tensile specimens whose length is parallel to the weft direction of the carbon-aramid reinforcement fabric. It can be noted that the longitudinal strains used to plot the experimental stress–strain curves shown in Figure 17 and Figure 18 were obtained by using a virtual extensometer monitored using the DIC method in tensile tests of the hybrid composite specimens.

In Table 8, the values of the equivalent tensile moduli of elasticity corresponding to the warp and weft directions of the reinforcement carbon-aramid fabric are synthesized. By comparing these values, the errors are computed and given in the last three columns of Table 8. The greatest values of the errors correspond to the comparison between the results obtained by means of FEA and the experimental results.

**Figure 19:**
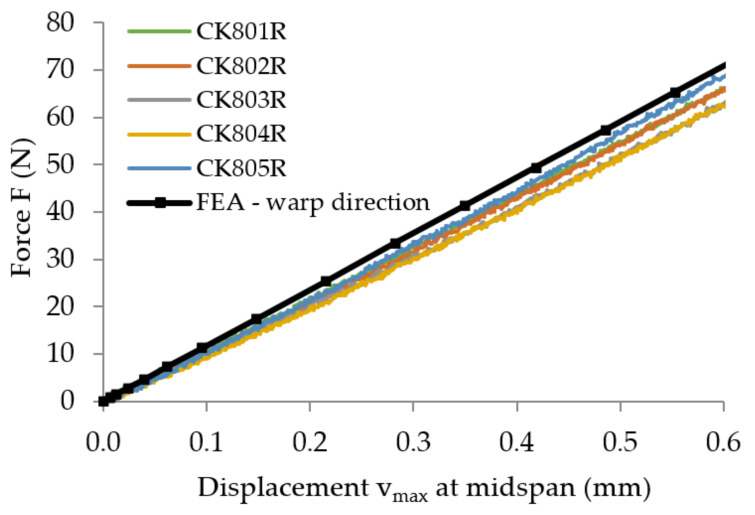
Comparison of the force–displacement curves obtained through the bending test of the flexural specimen parallel to the warp direction and the curve obtained by numerical simulation.

**Figure 20:**
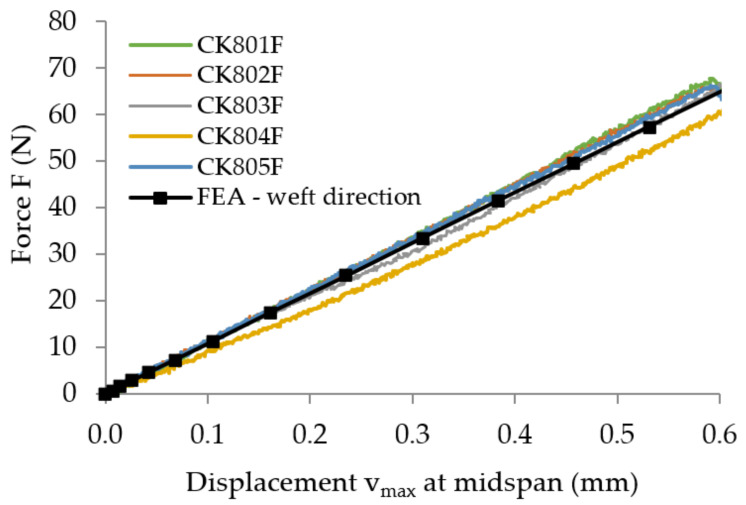
Comparison of the force–displacement curves obtained through the bending test of the flexural specimen parallel to the weft direction and the curve obtained by numerical simulation.

The curve of the bending force related to the maximum displacement, obtained by numerical simulation, is superimposed onto the curves recorded in bending tests for the two kinds of flexural specimen: where the specimen length is parallel to the warp direction of the reinforcement carbon-aramid fabric (Figure 19), and where the specimen length is parallel to the weft direction of the reinforcement carbon-aramid fabric (Figure 20).

The values of the equivalent flexural moduli of elasticity corresponding to the warp and weft directions of the reinforcement carbon-aramid fabric are synthesized in Table 9. It is again noted that the greatest values of the errors correspond to the comparison between the results obtained by means of FEA and the experimental results.

## 4. Conclusions

This paper reports the elastic constants including Poisson’s ratio and mechanical characteristics for the hybrid carbon-aramid/epoxy composite material, obtained by tensile testing combined with the DIC method, by the bending test and by Charpy impact tests. In order to determine the Poisson ratio in the reinforcement plane with carbon-aramid woven fabric, the curves of the transverse strains plotted as a function of the longitudinal strains are accurately determined by using the DIC method.

Analytical models are used to compute the tensile and flexural moduli of elasticity corresponding to the fictitious orthotropic material which behaves similarly to the hybrid carbon-aramid composite involved in this research, in tensile and bending tests, respectively.

Further, the elastic properties are used to model the laminated composite material having eight layers reinforced with carbon-aramid woven fabric, all layers having the same thickness. The numerical model is used for simulation of the distributions of the strains and stresses developed in tensile and bending tests of the specimens made of such hybrid composite material. Finally, it was observed that the experimental stress–strain curves recorded in tensile tests and force-displacement curves obtained in bending tests match very well with the curves obtained by numerical simulation. The numerical model is validated by the experiment, since the maximum errors recorded between experimental and theoretical results were 0.19% (Table 8) and 0.15% (Table 9) for the equivalent tensile modulus and for the equivalent flexural modulus, respectively. Moreover, the stress–strain curves obtained by means of FEA fit with stress–strain curves recorded in tensile tests (Figure 17 and Figure 18). The force–displacement curves obtained by numerical simulation also match with the curves recorded in bending tests for the linear deformations (Figure 19 and Figure 20). It follows that the elastic constants reported in this paper may be used in further research in order to simulate stresses and strains developed in mechanical structures made of the hybrid composite material involved in this investigation.

The numerical simulations of the hybrid carbon-aramid composite are limited to just the linear field of the material behaviour. Numerical simulations regarding the nonlinear behaviour of the carbon-aramid composite could be the subject of further research.

The full strain fields that occur in the bending and impact tests are not monitored using the DIC method in this research, because the thickness (2.6 mm) of the specimens is too small to obtain a good quality of the speckle pattern on the side surfaces of specimens. Moreover, the roughness of the side surfaces is not fine due to the jet cutting of test specimens. As a result, the authors intend to approach the monitoring using the DIC method of the bending and impact tests in their further research on the mechanical behaviour of carbon-aramid composites.

Another objective of further research is studying the mechanism of the hybridization effect of carbon fibres and aramid fibres on the mechanical properties of the hybrid composite materials reinforced with carbon-aramid woven fabric.

## Figures and Tables

**Table 1:** Tensile properties for carbon-aramid composite material.

Specimen Direction	Specimen Code *	Dimensions of Cross-Section		Young’s Modulus *E* (MPa)	Maximum Force *F*_max_ (N)	Tensile Strength σ_max_ (MPa)	Maximum Strain ε_max_ at *F*_max_	Poisson’s Ratio ν
		*b* (mm)	*h* (mm)					
Warp (R)	CK801R	10.20	2.60	35,882	10,775	406	0.0136	0.155
	CK802R	10.34	2.57	36,108	11,722	441	0.0117	0.119
	CK803R	10.33	2.66	36,574	10,434	380	0.0097	0.151
	CK804R	10.48	2.68	35,458	11,093	395	0.0108	0.122
	CK805R	10.46	2.78	32,205	12,013	413	0.0163	0.158
Average (stdev)				35,245 (1747)	11,207 (654)	407 (23)	0.0124 (0.0026)	0.141 (0.019)
Weft (F)	CK801F	10.60	2.74	33,721	11,127	383	0.0113	0.099
	CK802F	10.5	2.65	33,627	10,379	373	0.0107	0.0106
	CK803F	10.61	2.82	35,107	11,760	393	0.0101	0.118
	CK804F	10.52	2.74	33,928	9741	338	0.0102	0.106
	CK805F	10.6	2.78	31,747	11,016	374	0.0111	0.102
Average (stdev)				33,626 (1207)	10,805 (770)	372 (21)	0.0107 (0.0005)	0.106 (0.007)

* The last letter specimen code indicates the direction of the tensile specimen: R—warp direction; F—weft direction.

**Table 2:** Flexural properties for carbon-aramid composite material.

Specimen Direction	Specimen Code *	Dimensions of Cross-Section		Flexural Modulus of Elasticity *E* (MPa)	Maximum Force *F*_max_ (N)	Maximum Stress σ_max_ (MPa)	Maximum Displacement *v*_max_ at *F*_max_ (mm)
		*b* (mm)	*h* (mm)				
Warp (R)	CK801R	14.99	2.55	29,331	435	429	5.376
	CK802R	14.92	2.58	28,846	396	382	4.686
	CK803R	14.96	2.52	29,088	439	443	4.916
	CK804R	14.96	2.52	29,409	418	422	5.016
	CK805R	14.98	2.58	30,042	430	414	4.701
	Average (stdev)			29,343 (449)	424 (17)	418 (23)	4.939 (0.282)
Weft (F)	CK801F	15.06	2.72	24,899	377	325	5.400
	CK802F	14.88	2.56	29,557	357	351	5.106
	CK803F	14.98	2.7	24,767	412	362	4.435
	CK804F	14.93	2.61	27,456	356	336	5.354
	CK805F	14.85	2.61	27,741	413	392	5.222
	Average (stdev)			26,884 (2039)	396 (28)	363 (26)	5.106 (0.391)

* The last letter specimen code indicates the direction of the tensile specimen: R—warp direction; F—weft direction.

**Table 3:** Results obtained by Charpy impact testing.

Direction of the Specimen	Impact Failure Energy W (J)	Impact Strength *K* (kJ/m^2^)
Warp direction (R)	2.79 (0.23) *	90.49 (5.88) *
Weft direction (F)	2.43 (0.12) *	80.36 (3.19) *

* The values shown in the brackets represent stdev values.

**Table 4:** Characteristics of the carbon-aramid layer computed by using the analytical method and considering the results obtained in tensile tests.

Direction	Thickness $t_{k}$ of the Layer (mm)	Elastic Characteristics of the Layer			Terms of the Stiffness Matrix ${\left[Q\right]}_{k}$ of the Composite Layer		
		$E_{1}$ (MPa)	$E_{2}$ (MPa)	$\nu_{12}$	$Q_{11}$ (MPa)	$Q_{12}$ (MPa)	$Q_{22}$ (MPa)
Warp (R)	0.325	35,245	33626	0.141	35,927.625	4833.095	34,277.268
Weft (F)		33,626	35245	0.106	34,027.525	3780.581	35,665.857

**Table 5:** Characteristics of the carbon-aramid layer computed by using the analytical method and considering the results obtained in bending tests.

**Direction**	Thickness $t_{k}$ of the Layer (mm)	Elastic Characteristics of the Layer			Terms of the Stiffness Matrix ${\left[Q\right]}_{k}$ of the Composite Layer		
		$E_{1}$ (MPa)	$E_{2}$ (MPa)	$\nu_{12}$	$Q_{11}^{\prime }$ (MPa)	$Q_{12}^{\prime }$ (MPa)	$Q_{22}^{\prime }$ (MPa)
Warp (R)	0.325	29,343	26,884	0.141	29,886.942	3860.913	27,382.359
Weft (F)		26,884	29,343	0.106	27,218.791	3149.092	29,708.413

**Table 6:** Stiffness matrix components and equivalent moduli of elasticity of the carbon-aramid laminated composite material, computed by using the analytical method.

**Direction**	Stiffness Matrix Components						Equivalent Tensile Modulus of Elasticity *E* (MPa)	Equivalent Flexural Modulus of Elasticity *E’* (MPa)
	$A_{11}$ (MPa)	$A_{12}$ (MPa)	$A_{22}$ (MPa)	$D_{11}$ (MPa)	$D_{12}$ (MPa)	$D_{22}$ (MPa)		
Warp (R)	93,411.83	12,566.05	89,120.90	43,774.41	5654.95	40,106.03	33,626.78	29,342.55
Weft (F)	88,471.57	9829.51	92,731.23	39,866.46	4612.37	43,512.92	35,246.16	26,884.99

**Table 7:** Comparison between the results obtained using the DIC method and by means of FEA concerning the longitudinal strains $\varepsilon_{l}$ for a tensile specimen loaded in the warp direction.

No.	Normal Stress σ (MPa)	Longitudinal Strain $\varepsilon_{l}$		Err for $\varepsilon_{l}$ (%)
		DIC Method	FEA	
1	148.30	0.003801	0.004205	9.61
2	199.46	0.005276	0.005656	6.72
3	251.54	0.006568	0.007133	7.92
4	302.67	0.008245	0.008583	3.93
5	351.12	0.009486	0.009957	4.73
6	398.11	0.010752	0.011289	4.76

**Table 8:** Comparison of the results obtained for the tensile moduli of elasticity in the case of tensile loading.

Direction	Equivalent Tensile Modulus of Elasticity $E_{t}$ (MPa)			Error (%)		
	AnM *	FEA	Exp *	FEA vs. AnM	Exp vs. AnM	Exp vs. FEA
Warp (R)	35,246.16	35,311	35,245	0.18	0.003	0.19
Weft (F)	33,626.78	33,684	33,626	0.17	0.002	0.17

* AnM—analytical method; Exp—experimental results.

**Table 9:** Comparison of the results obtained for the flexural moduli of elasticity in case of bending loading.

Direction	Equivalent Flexural Modulus E′ (MPa)			Error (%)		
	AnM *	FEA	Exp *	FEA vs. AnM	Exp vs. AnM	Exp vs. FEA
Warp (R)	29,342.55	29,387.40	29,343.00	0.15	0.002	0.15
Weft (F)	26,884.99	26,877.70	26,884.00	0.03	0.004	0.02

* AnM—analytical method; Exp—experimental results.

## Data Availability

Not applicable.
